# Cost-effectiveness analysis of ceritinib vs. crizotinib in previously untreated anaplastic lymphoma kinase (ALK)-positive non-small cell lung cancer (NSCLC) in Hong Kong

**DOI:** 10.1186/s12962-020-00244-6

**Published:** 2020-11-07

**Authors:** Herbert H. Loong, Carlos K. H. Wong, Linda K. S. Leung, Catherine P. K. Chan, Andrea Chang, Zheng-Yi Zhou, Jipan Xie, Meaghan Gibbs

**Affiliations:** 1 Department of Clinical Oncology, The Chinese University of Hong Kong, Hong Kong, China; 2 Department of Family Medicine and Primary Care, The University of Hong Kong, Hong Kong, China; 3 Novartis Pharmaceuticals Corporation, Hong Kong, China; 4 Analysis Group, Inc, London, UK; 5 Analysis Group, Inc, Los Angeles, CA USA; 6 Novartis Pharmaceuticals Corporation, Dubai, United Arab Emirates

**Keywords:** Advanced non-small cell lung cancer, Anaplastic lymphoma kinase-positive, Ceritinib; cost-effectiveness, Hong kong

## Abstract

**Introduction:**

Lower-dose ceritinib (450 mg) once-daily with food was approved in 2018 in Hong Kong (HK) for first-line treatment of patients with anaplastic lymphoma kinase-positive (ALK +) advanced non-small cell lung cancer (NSCLC). This study examined the cost-effectiveness of ceritinib vs. crizotinib in the first-line treatment of ALK + NSCLC from a HK healthcare service provider's or government's perspective.

**Methods:**

Costs and effectiveness of first-line ceritinib vs. crizotinib over a 20-year time horizon was evaluated using a partitioned survival model with three health states (stable disease, progressed disease, and death). The efficacy data for ceritinib were obtained from a phase 3 trial comparing ceritinib with chemotherapy for advanced non-small cell lung cancer (ASCEND-4) and extrapolated using parametric survival models. Long-term survival associated with crizotinib were estimated using hazard ratio of crizotinib vs. ceritinib obtained from matching-adjusted indirect comparison based on ASCEND-4 and PROFILE 1014 trials. Drug acquisition, administration, adverse events costs, and medical costs associated with each health state were obtained from public sources and converted to 2018 US Dollars. Incremental costs per quality-adjusted-life-year (QALY) and life-year (LY) gained were estimated for ceritinib vs. crizotinib.

**Results:**

The base case results showed that ceritinib was associated with 3.22 QALYs, 4.51 LYs, and total costs of $157,581 over 20 years. Patients receiving crizotinib had 2.68 QALYs, 3.85 LYs, and $150,424 total costs over the same time horizon. The incremental cost per QALY gained for ceritinib vs crizotinib was $13,343. Results were robust to deterministic sensitivity analyses in most scenarios.

**Conclusion:**

Ceritinib offers a cost-effective option compared to crizotinib for previously untreated ALK + advanced NCSLC in HK.

## Introduction

The discovery of different genomic mutations involving in the growth and progression of non-small-cell lung cancer (NSCLC) has led to the development of novel targeted treatments to specific gene mutations. Anaplastic lymphoma kinase (ALK) rearrangements, also known as EML4-ALK fusion, are mutations to the ALK gene and account for lung carcinogenesis in around 5% of patients with NSCLC [1].

Crizotinib was the first ALK inhibitor approved in Hong Kong (HK) to treat patients with ALK + advanced NSCLC. However, the response to crizotinib varies and some patients, particularly those with brain metastases, are less likely to benefit as crizotinib may insufficiently cross the blood–brain barrier, leaving metastases in the brain inadequately treated [2]. This is a key limitation of crizotinib as the control of brain metastases is an important therapeutic goal given that the central nervous system is a common site of disease in advanced NSCLC. Moreover, patients with an initial response to crizotinib develop resistance to crizotinib within 1–2 years [3, 4].

Ceritinib, a second-generation ALK inhibitor, was approved in HK in September 2017 for the first-line treatment of patients with ALK + advanced NSCLC [5]. Results from ASCEND-4, a phase III trial, showed a significant improvement in median progression free survival (PFS) for ceritinib 750 mg treatment compared to platinum doublet chemotherapy (16.6 months vs. 8.1 months) [5]. The ASCEND-8 bioequivalence trial showed similar pharmacokinetics and better gastrointestinal safety profile of lower dose ceritinib − 450 mg with food, versus ceritinib 750 mg [6, 7] Ceritinib 450 mg with food was approved in HK for the first-line treatment of patients with ALK + advanced NSCLC in May 2018.

In the first-line setting, crizotinib is one of several ALK inhibitors for lung cancer covered by the publicly funded Samaritan Fund in HK [8]. However, with the advent of ceritinib as a new first-line treatment option, it is important for stakeholders to understand whether this new treatment represents long-term value for money compared to crizotinib in HK. Such insight has the potential to help healthcare stakeholders optimize treatment regimens for patients with NSCLC. In order to help fill this gap in knowledge, this study examined the cost-effectiveness of first-line ceritinib compared to crizotinib in the treatment of patients with ALK + advanced NSCLC in HK.

## Methods

### Model structure

**Fig. 1:**
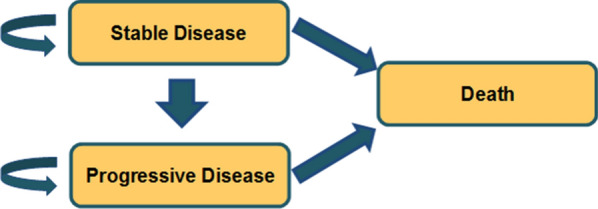
Partitioned survival model structure

A partitioned survival model was developed in Microsoft Excel 2016 (Microsoft Corp., Redmond, VA) to track the disease course and survival of adults with advanced ALK + NSCLC who had not previously received treatment with systemic anti-cancer therapy. Partitioned survival analysis is a common approach in modeling advanced or metastatic cancers and has previously been used in many cost-effectiveness analyses (CEA) in a NSCLC setting [9–14]. The approach uses clinical endpoints in trials to estimate the distribution of patients across various health states over time using the area under the survival curves. The model comprised three mutually exclusive health states: (1) stable disease; (2) progressed disease; and (3) death (Fig. 1). The proportion of patients in the progressed state at each cycle was calculated as the difference between the proportion of patients surviving (i.e., OS) and the proportion of patients remaining in the stable state (i.e., PFS).

At model entry, all patients were in the stable disease (starting age: 54 years), which reflected the median age of the patient population in ASCEND-4 [5]. In any of the following cycles, patients either stayed in the same health state or transitioned to a progressive disease state or death, which was the absorbing state.

The model compared ceritinib 450 mg orally once daily to crizotinib 250 mg orally twice daily, both are currently licensed dosages for the treatment of patients with ALK + NSCLC in HK. Patients were modeled with 1-month cycles over a 20-year time horizon which comprehensively captures the expected costs and health outcomes of patients over their remaining lifetime from first-line treatment initiation. In the base case analysis, both costs and effectiveness were discounted at a rate of 3.5% per year, as recommended by the National Institute of Health and Care Excellence (NICE) in the UK. The model was developed from a perspective of healthcare service providers or the government, thus only direct healthcare costs were considered.

### Model assumptions

Due to the limited follow up and difference in primary endpoint of ASCEND-8, [6] the efficacy and safety data for the ceritinib arm were based on ASCEND-4, [5] which studied the efficacy of ceritinib 750 mg orally. The ASCEND-8 trial showed that ceritinib 450 mg, taken once daily with food demonstrated comparable pharmacokinetics as daily dose of 750 mg in a fasted state. Therefore, the efficacy of ceritinib 750 mg and 450 mg were assumed the same based on the ASCEND-8 data, regulatory agencies (e.g. Food and Drug Administration, European Medicines Agency, HK Department of Health) have replaced the 750 mg (in a fasted state) with 450 mg (taken with food) as the standard dose for ceritinib. Patients in the stable disease state incurred drug and drug administration costs, a one-off treatment-associated adverse event (AE) cost, and medical costs associated with resource use. Patients in the progressed disease state incurred medical costs and additional costs of second-line treatments, which reflected those observed in the respective trials [5, 15]. All patients incurred one-off terminal care costs before death, irrespective of whether the death was related to NSCLC. Health utilities were dependent on health state, and additionally, the utility value for stable disease depended on the first-line treatment received. Thus, disutilities associated with AEs were not considered to avoid double counting. Finally, ALK testing was assumed to be a routine diagnostic test in HK and was therefore not considered as a cost component in the analysis.

### Model inputs

#### Efficacy inputs

Time spent in the stable disease, progressed disease, and death states were estimated for both ceritinib and crizotinib arms based on respective areas under the PFS and overall survival (OS) curves during the model’s 20-year time horizon.

For ceritinib, survival curves were estimated and extrapolated beyond the trial period by fitting parametric models to individual patient level data for PFS and OS from the ASCEND-4 trial [5]. Proportional hazards models based on Weibull, exponential, and Gompertz distributions were applied based on the methods recommended by the NICE decision support unit [16]. Selection of base-case models was informed by comparisons of goodness-of-fit criteria, evaluation of log-cumulative hazard plots, and expert opinion on the clinical plausibility of long-term outcome predictions. For PFS, while the Gompertz function demonstrated the best fit based on AIC/BIC, the long-term extrapolation using the exponential function were considered to be more clinically plausible. For OS, exponential curve demonstrated the best fit to match the observed OS data and the log-cumulative hazard plot was also linear in shape. Based on that, the exponential function was selected as the base-case parametric model for both PFS and OS and other parametric proportional hazards models (e.g., Weibull, Gompertz) were explored in the sensitivity analysis.

**Fig. 2:**
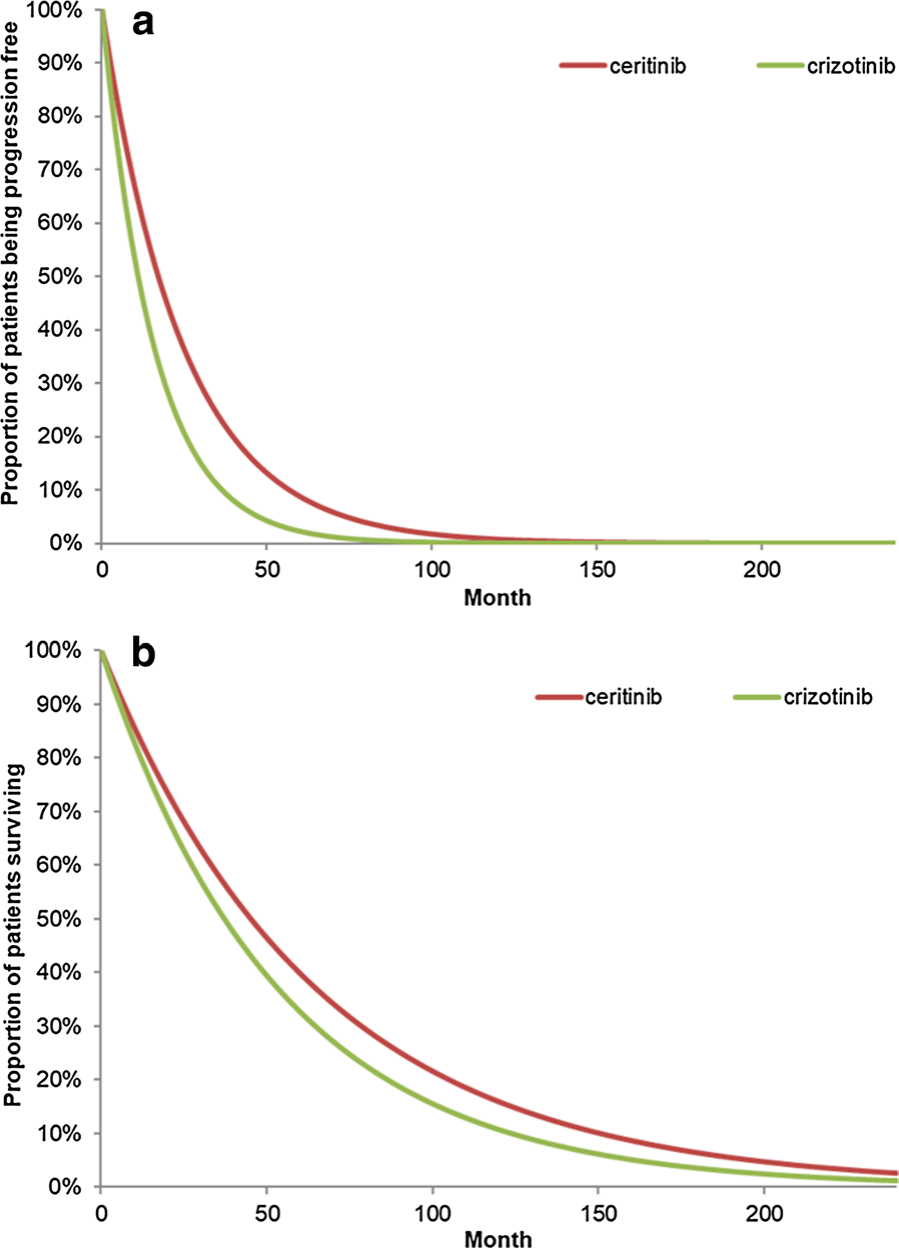
**a** Predicted PFS^*^, **b** OS^†^ for Ceritinib and Crizotinib under HR Method, ^*^The predicted PFS curve for ceritinib was based on exponential functions. PFS curve for crizotinib was derived by applying HRs to the corresponding curve for ceritinib. ^†^The predicted OS curve for ceritinib was based on exponential functions. OS curve crizotinib was derived by applying HRs to the corresponding curve for ceritinib. HR, hazard ratio; PFS, progression-free survival; OS, overall survival

For crizotinib, PFS and OS were extrapolated by applying the corresponding hazard ratios (HRs) for crizotinib vs. ceritinib to the PFS and OS parametric models of the ceritinib arm. In the absence of head-to-head randomized trials comparing ceritinib to crizotinib in previously untreated advanced or metastatic ALK + NSCLC, an indirect comparison needed to be considered. Although the patient population enrolled in ASCEND-4 and PROFILE 1014 trials and both trials included chemotherapy as a comparator arm, the chemotherapy arms were significantly different to be used as a common comparator. Therefore, ‘anchor-based’ analysis was not feasible and a matching-adjusted indirect comparison (MAIC) in a non-anchor based setting was used to compare efficacy outcomes between crizotinib and ceritinib [17]. The MAIC indirectly compared the ceritinib arm from ASCEND-4 [5] to the crizotinib arm from PROFILE 1014, [15] while adjusting for cross-trial differences in patient characteristics between the two trials. Compared to crizotinib, ceritinib was associated with a significantly longer PFS [HR = 0.64; 95% confidence interval (CI) 0.47–0.87] and median PFS of 25.2 vs 10.8 months; the two treatments were similar with respect to OS 0.82; 95% CI 0.54, 1.27) (Table 1) [17]. Predicted PFS and OS for both treatments are shown in Fig. 2.

**Table 1:** Summary of model inputs

Model input	Value	Notes/sources
Utility inputs for disease states		
Stable disease: ceritinib	0.81	ASCEND-4 (Soria et al. [5])
Treatment response: Ceritinib	0.81	
Stable disease: crizotinib	0.81	Felip et al. [22, 26]
Treatment response: Crizotinib	0.81	
Disease progression: all treatments	0.64	Chouaid et al. [27]
Hazard ratios		
PFS: Ceritinib vs. Crizotinib	0.64 (0.47, 0.87)	Li et al. [17]
OS: Ceritinib vs. Crizotinib	0.82 (0.54, 1.27)	
Unit cost		
Ceritinib (450 mg)	$52.77 per pill	Hong Kong Hospital Authority [1]
Crizotinib (250 mg)	$113.21 per pill	
Relative dose intensity		
Ceritinib	92.2%	ASCEND-8 [6]
Crizotinib	92.0%	PROFILE 1014 (Australian Department of Health) [15]
Monthly medical costs/ pre-progression frequency/post-progression frequency		
Cancer nurse	$12.82/ 0.1 time per month/0.1 time per month	Hong Kong Hospital Authority^a^
Outpatient visit	$142.31/ 1 time per month / 1.08 times per month	
Pharmacist	$49.36/ 1 time per month /0.1 time per month	
Complete blood count	$17.31 / 1 time per month / 1.08 times per month	
CT scan	$1,730.77/ 0.25 time per month / 0.18 time per month	
MRI	$602.56/ 0.17 time per month /0.12 time per month	
X-Ray	$24.36/ 1 time per month / 1.08 times per month	
Serum chemistry	$116.03/ 1 time per month / 1.08 times per month	
Total post-progression treatment costs		
Ceritinib	$11,142.92	ASCEND-4; PROFILE 1014 [15]; Hong Kong Hospital Authority [1] and literature
Crizotinib	$10,385.68	
Terminal care cost	$24,089.00	Wong et al. [25]
Costs associated with AEs		
Ceritinib	$2,024.93	ASCEND-8 (Cho et al. [6] PROFILE 1014 (Solomon et al. [15]) Hong Kong Hospital Authority [1]
Crizotinib	$1,236.39	

AE, adverse event; CT, computed tomography; MRI, magnetic resonance imaging; OS, overall survival; PFS, progression-free survival

All costs were converted to 2018 US Dollars (USD) from Hong Kong Dollars (HKD) using a prevailing exchange rate obtained from the Linked Exchange Rate System in Hong Kong (1 USD = 7.8 HKD)

^a^Hong Kong Monetary Authority. Fees and Charges. https://www.ha.org.hk/visitor/ha_visitor_index.asp?Content_ID=10045&Lang=ENG Accessed January 10, 2020

#### Resource use and cost inputs

All costs were converted to 2018 US Dollars (USD) from Hong Kong Dollars (HKD) using a prevailing exchange rate obtained from the Linked Exchange Rate System in Hong Kong (1 USD = 7.8 HKD) [18].

##### Cost of first-line treatment

Monthly drug costs for ceritinib and crizotinib were estimated based on local unit drug costs, the licensed dosage, relative dose intensity and duration of treatment. Costs per capsule for ceritinib and crizotinib were obtained from the Hong Kong Hospital Authority databases [19]. Mean relative dose intensity for ceritinib (450 mg) were based on the ASCEND-8 trial [6, 15] Because the relative dose intensity for crizotinib was not reported in PROFILE 1014, it was obtained from PROFILE 1007, a Phase III open-label trial for crizotinib among previously treated patients with ALK + NSCLC [20]. No administration costs were assumed for oral drugs.

Patients were assumed to be on first-line treatment until discontinuation or progression, whichever occurs first. The proportion of patients on treatment was estimated based on PFS multiplied by the ratio of the proportion of patients remaining on treatment and the proportion of patients in PFS, which was estimated for each cycle using patient-level data from the ASCEND-4 trial [5]. For crizotinib, the ratio was assumed to be the same as that of ceritinib.

##### Cost of subsequent therapy.

Post-progression treatment costs with active agents (e.g., ceritinib, crizotinib, docetaxel, and platinum based chemotherapy [21]) were applied to both treatment arms for patients who newly entered the progressed disease state in each model cycle. The model assumed that 20% of post progression patients did not receive any further systemic therapy due to rapid performance deterioration or death. Among patients who initiated systemic second-line therapy after progression, the distribution of patients across second-line treatment options was estimated based on the relative frequency of different second-line treatments observed in ASCEND-4 and PROFILE 1014 trials [5, 15]. Drug unit cost, drug administration cost, and dosing schedule for chemotherapy were obtained from the Hong Kong Hospital Authority databases [19]. Mean relative dose intensity and treatment duration for each treatment option were obtained from trials in previously treated ALK + population [20, 22]. Body surface area (BSA) used to estimate required chemotherapy doses was based on clinical practice experience by the investigators [23].

##### Cost of AEs

Grade 3/4 AEs were included if they were reported in ≥ 5% of patients for at least one treatment in the model. The rates of AEs were based on rates reported in the ASCEND-8 trial for ceritinib, [6] and the PROFILE 1014 trial for crizotinib [15] Patients were assumed to incur a one-time cost for the management of AEs. AE management procedures were based on the clinical opinion of the investigators. The costs of AEs were obtained from the list of charges of the Hong Kong Hospital Authority [24].

##### Disease management cost by health state

Medical costs incurred during stable and progressed disease states were determined by monthly frequencies based on expert opinion and unit costs from the Hong Kong Hospital Authority databases [24]. Resource use during stable disease included visits to cancer nurses, pharmacists and outpatient visits. Tests and procedures performed included complete blood counts, computed tomography scans, magnetic resonance imaging, X-rays, and serum chemistry testing on a regular basis. In the progressed disease state, resource use for cancer nurse visits, pharmacist visits, outpatient visits, palliative medications, and home oxygen were included as well as all other tests and procedures from the pre-progression state. With respect to terminal care costs, all patients who transitioned to death were assumed to incur a one-time terminal care cost of $24,089 [25] This figure was based on a study of cost effectiveness of biennial mammography in women HK, that estimated terminal care cost during the 6 months before death [25].

#### Utility inputs

In the base case, utilities associated with stable disease for ceritinib were based on EQ-5D data from the ceritinib arm in the ASCEND-4 trial [5] and the utilities for crizotinib were obtained from an analysis of EQ-5D data from PROFILE 1014 reported by Felip et al. [26] For utilities in the progressed disease state, EQ-5D scores were not collected systematically after treatment discontinuation in ASCEND-4 or PROFILE 1014 trials. Given this lack of information, for all treatment arms, the base-case utility value for progressive disease (0.64) was estimated based on the utility study by Chouaid et al. [27]. To avoid double-counting the impact of AEs on quality of life, AE disutilities were not applied.

### Model outputs

Total costs and effectiveness outcomes were estimated for each arm during the 20-year model time horizon. Effectiveness outcomes included total life years (LYs), and quality-adjusted life years (QALYs). The incremental cost-effectiveness ratio (ICER) of ceritinib compared to crizotinib was evaluated using incremental cost per QALY gained and incremental cost/LY gained.

### Sensitivity analyses

**Fig. 3:**
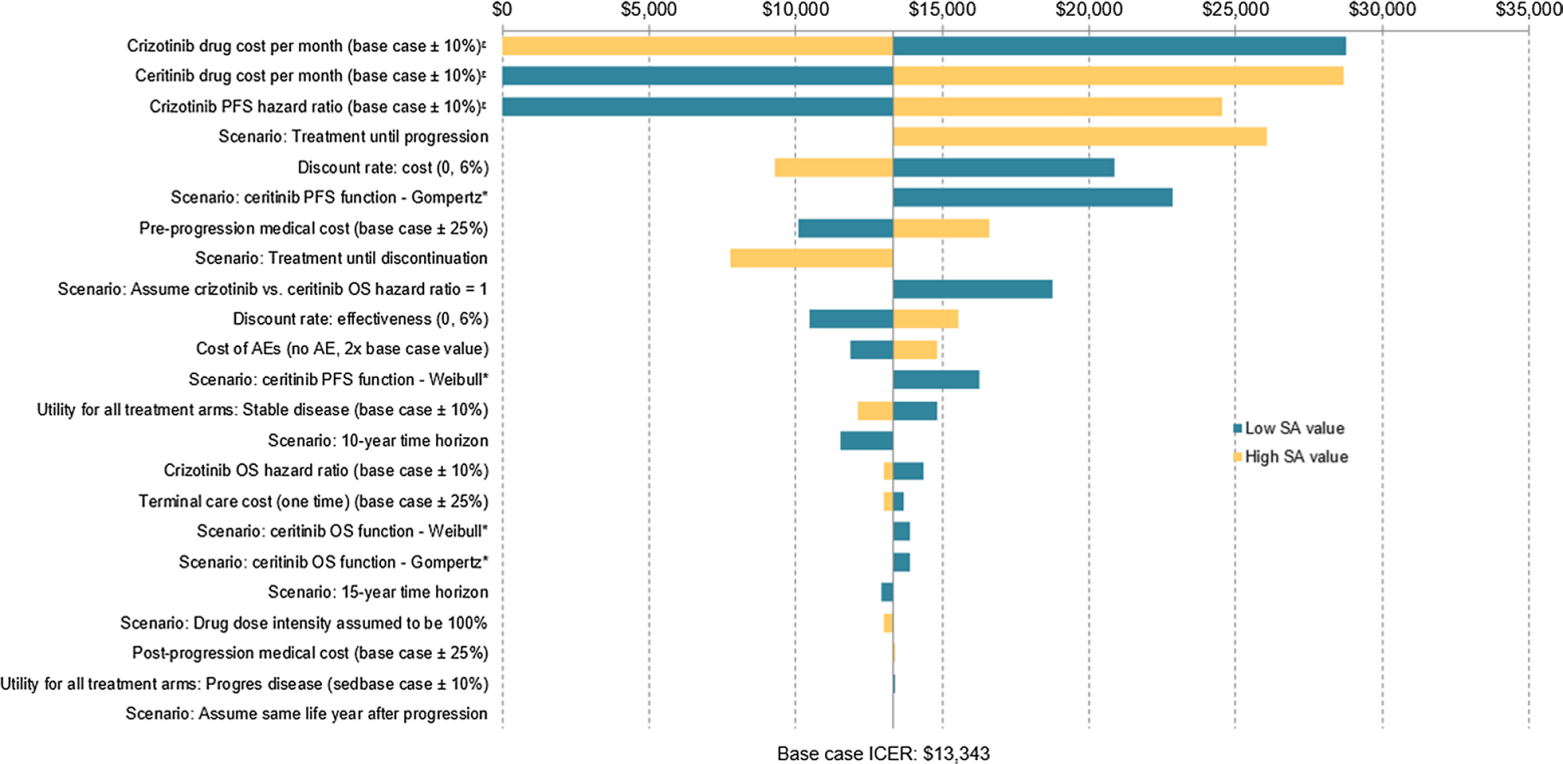
DSA Results for Ceritinib vs. Crizotinib. AE, adverse event, ICER, incremental cost-effectiveness ratio; PFS, progression-free survival; OS, overall survival

A deterministic sensitivity analysis (DSA) was performed to test the robustness of the model. The model uncertainty was assessed by varying one model input or assumption at a time. These included drug cost unit cost, efficacy for both arm, relative dose intensity, resource use, utility, discount rate, and time horizon. The detailed list of DSA inputs and their variation are illustrated in Fig. 3.

## Results

### Base case results

Over a 20-year time horizon, ceritinib 450 mg indicated greater health and quality of life benefits (more LYs and QALYs) versus crizotinib, at a lower cost during initial treatment. First-line treatment with ceritinib 450 mg was associated with 0.66 additional LYs (ceritinib: 4.51 LYs, crizotinib: 3.85 LYs) and 0.54 additional QALYs (ceritinib: 3.22 LYs, crizotinib: 2.68 LYs) compared to crizotinib, while the incremental costs for initial treatment was −$408 US dollars (USD) (cost saving). Overall, ceritinib 450 mg was associated with total direct costs of $157,581 USD and crizotinib was associated with $150,424 USD after discounting. The incremental costs of ceritinib vs. crizotinib was $7,157 USD. The greatest component of the difference in incremental costs of ceritinib vs crizotinib was medical costs ($6,309 USD) (Table 2).

**Table 2:** Base case results

	Ceritinib (450 mg)	Crizotinib	Ceritinib vs. Crizotinib
Cost (USD)			
Drug and drug administration costs, initial treatment	82,215	82,623	− 408
Drug and drug administration costs, post-progression treatment	10,392	9,921	471
Treatment associated adverse event costs	1,982	1,197	785
Medical costs	62,992	56,683	6,309
Total costs	157,581	150,424	7,157
Effectiveness			
Total QALYs	3.22	2.68	0.54
Total LYs	4.51	3.85	0.66
Incremental cost-effectiveness ratio (ICER) (USD)			
Incremental cost per QALY gained			13,343
Incremental cost per LY gained			10,794

ICER, incremental cost-effectiveness ratio; LY, Life-year; mg, milligram; QALY, quality-adjusted life year; USD, United States Dollar

The incremental cost/QALY gained for ceritinib as a first-line treatment for ALK + NSCLC over a 20-year time horizon was estimated to be $13,343 USD/QALY compared with crizotinib. The incremental cost/LY for ceritinib vs. crizotinib was estimated to be $10,794 USD/LY gained (Table 2).

### Sensitivity analyses

The DSA results showed that the ICER for ceritinib vs. crizotinib was robust in most scenarios. The ICER for ceritinib vs. crizotinib ranged from dominant (better QALY, lower costs) to $28,746/QALY when crizotinib unit price was decreased by 10%. The results were most sensitive to the monthly drug costs for crizotinib and ceritinib, HR ratio of PFS for crizotinib vs. crizotinib, and assumption on treatment until progression (Fig. 3).

## Discussion

This study provides the first estimate of the cost-effectiveness of ceritinib compared to crizotinib in adult patients with previously untreated ALK + NSCLC in HK from a healthcare service provider’s or government’s perspective. With ceritinib's recent introduction as a first-line targeted therapy, these results provide important insight for decision-makers considering treatments for ALK + advanced NSCLC in HK.

The results from this economic evaluation suggested that over a 20-year time horizon, ceritinib conferred greater health benefits with a gain of 0.54 additional QALYs and $7,157 higher costs compared to crizotinib. The corresponding incremental cost/QALY gained over a 20-year time horizon was estimated to be $13,343/QALY for ceritinib vs. crizotinib. The ICER is significantly lower than the widely used World Health Organization (WHO) threshold of three times gross domestic product (GDP)/capita, which is currently at $119,274 USD in HK [28]. Therefore, the study’s findings support ceritinib as a cost-effective option in the treatment of previously untreated ALK + advanced NSCLC compared to crizotinib in HK. The results were robust in sensitivity analyses, including analysis based on varying utility values from ASCEND-4, PROFILE 1014 and additional literature (ICER of $14,818/QALY), as well as varying discount rates (ICER of $20,868/QALY) and treatment until progression ($26,052/QALY).

Without a head-to-head trial, MAIC—an indirect comparison method was used to indirectly compare efficacy outcomes between crizotinib and ceritinib, while adjusting for differences between trials in baseline patient characteristics [29]. The MAIC methodology is an extension of propensity score weighting, which has long been used in epidemiological studies for adjusted comparisons of non-randomized treatment groups [30, 31]. Without a connected network of randomized controlled studies, or where there are single-arm studies involved, MAIC is one of the recommended methods for population-adjusted indirect comparison [32] and has been increasingly used in HTA submissions [33]. Without a head-to-head comparison of two treatments, MAIC may be the best available evidence. However, the inherited methodological limitations (e.g., unobserved differences between the trials) and impact on the results due to immature follow-up should be noted. In this study, the uncertainty in the MAIC estimates was explored in the sensitivity analysis. Increasing and decreasing the HR for OS by 10% results in the ICER varying between $10,003 USD and $14,339 USD (3% lower and 7% higher, respectively). When assuming the same OS for ceritinib and crizotinib, the ICER increased to $18,757 USD (41% higher), which is still considered cost effective.

Prior cost-effectiveness studies comparing ceritinib to crizotinib in patients with ALK + NSCLC have largely focused on previously treated patients [34–37]. One study in the US that examined the cost-effectiveness of ceritinib compared to crizotinib in previously untreated patients with ALK + NSCLC based on efficacy using indirect treatment comparison. The incremental cost per QALY gained was $66,064 for ceritinib versus crizotinib, showing ceritinib to be a more cost-effective treatment option in first line [38]. However, differences exist in the healthcare systems between the US and HK populations, which may translate into different resource utilization and disease management. More recently, a CEA of ceritinib and alectinib versus crizotinib was conducted from the Chinese healthcare perspective [39]. In the study, ceritinib yielded an additional 1.09 QALYs and incremental costs of $15,165 compared with crizotinib, resulting in an ICER of $13,905 per QALY, which is similar to what we found from the HK perspective.

Considering the limitations associated with crizotinib therapy in terms of resistance and inadequate effect on brain metastases, ceritinib offers a favorable treatment option for first-line treatment of ALK + NSCLC with the potential to improve outcomes and prolong survival.

This study should be interpreted in the context of certain limitations. First, PFS and OS beyond the trial period were based on extrapolation of survival curves using parametric models. The model’s results should be validated against long-term efficacy data from the trials or real-world evidence as the data become available. Second, due to the short follow up duration in ASCEND-8, while the dosing for ceritinib was based on ASCEND-8, the efficacy data was mainly from ASCEND-4, supported by the bioequivalence evidence. Neither trials were based exclusively on an Asian population, thus population-based differences in response and clinical outcomes may exist. There is evidence to suggest Asian patients achieved higher response rate to ALK inhibitors, compared to non-Asian patients, which may impact the survival outcomes and cost effectiveness results [40, 41]. Similarly, the utility values used were based on the UK population and not HK, due to the lack of data. Finally, MAIC was used in the absence of a head-to-head trial; residual biases due to unobserved effect modifiers and prognostic characteristics may exist.

## Conclusion

The greater LYs and QALYs as well as the longer PFS associated with ceritinib vs. crizotinib suggests that ceritinib has the potential to offer greater clinical benefits to patients. In addition, ceritinib appears to be highly cost-effective compared to crizotinib for previously untreated ALK + advanced NSCLC in HK.

## New knowledge added by this study

- In order to optimize patient outcomes and effectively allocate healthcare resources, rigorous economic evaluations are needed to inform reimbursement decision-making regarding the cost-effectiveness of available therapies.
- With ceritinib's recent introduction as a first-line targeted therapy, these results provide important insight for decision-makers considering treatments for ALK + advanced NSCLC in Hong Kong.

## Implication for clinical practice or policy

- Ceritinib offers a cost-effective option compared to crizotinib for previously untreated ALK + advanced NCSLC in HK with an incremental cost per QALY of $13,343 USD, which is significantly lower than the willingness to pay threshold in Hong Kong which is currently $119,274.
- In previously untreated adult patients with ALK + advanced NCSLC, ceritinib is predicted to offer marked benefits in PFS, LYs, and QALYs compared to crizotinib.
- Ceritinib offers a valuable treatment option for front-line treatment of patients with ALK + advanced NCSLC in HK.

## Data Availability

The datasets used and/or analysed during the current study are available from the corresponding author on reasonable request.
